# An attempt to explain the neurological symptoms of Myalgic Encephalomyelitis/Chronic Fatigue Syndrome

**DOI:** 10.1186/s12967-021-03143-3

**Published:** 2021-11-22

**Authors:** Klaus J. Wirth, Carmen Scheibenbogen, Friedemann Paul

**Affiliations:** 1 KOSA Pharma GmbH, Frankfurt am Main, Germany; 2 Institute of Medical Immunology, Charité - Universitätsmedizin Berlin, Corporate Member of Freie Universität Berlin, Humboldt-Universität zu Berlin, and Berlin Institute of Health, Berlin, Germany; 3 Experimental and Clinical Research Center, Max Delbrück Center for Molecular Medicine and Charité Universitätsmedizin Berlin, Berlin, Germany

**Keywords:** Chronic Fatigue Syndrome, Myalgic Encephalomyelitis, Post-Covid-19 syndrome, Neurological symptoms, Cognitive impairment, Brain fog, Hypersensitivity, Dysautonomia, Sleep disturbance, Headache

## Abstract

There is accumulating evidence of endothelial dysfunction, muscle and cerebral hypoperfusion in Myalgic Encephalomyelitis/Chronic Fatigue Syndrome (ME/CFS). In this paper we deduce the pathomechanisms resulting in central nervous pathology and the myriad of neurocognitive symptoms. We outline tentative mechanisms of impaired cerebral blood flow, increase in intracranial pressure and central adrenergic hyperactivity and how they can well explain the key symptoms of cognitive impairment, brain fog, headache, hypersensitivity, sleep disturbances and dysautonomia.

## Introduction

Myalgic Encephaloymelitis (ME/CFS) or Chronic Fatigue Syndrome is a frequent and debilitating but still enigmatic disease. It presents with a confusing variety of symptoms ranging from neurological symptoms, fatigue, exertional intolerance with post-exertional malaise (PEM), chronic muscle pain, skeletal muscle and cardiovascular findings to complaints arising from many other organs [11]. No effective treatment exists so far. ME/CFS is often triggered by infections with various viruses, like EBV, enteroviruses, influenza virus, dengue fever and as a recent example Corona virus (SARS-CoV2) [21, 22]. Very recent studies into post-Covid-19 syndrome (PCS) suggest that there is a broad overlap in symptomatology between both conditions [21, 25, 28, 29, 36, 45].

ME/CFS is classified as a neurological disease. This is based on neurological symptoms including mental fatigue, impaired cognition, psychomotor slowing, disturbed sleep, hypersensitivities to noise, light and smells, headache, pain and paresthesias and severe dysautonomia.

Many symptoms are, however, not obviously explained by neurological pathology including the cardiovascular situation with orthostatic intolerance, hypovolemia and a low activity of the renin–angiotensin–aldosterone system (RAAS), or the impaired muscle function (reduced handgrip strength and fatigability) and energetic disturbance. This co-occurrence of seemingly unrelated symptoms and findings prompts to look for a unifying explanation (the most parsimonious explanation).

In a first hypothesis paper initially stimulated by the finding in ME/CFS patients of autoantibodies against ß_2_-adrenergic receptors (ß2AdR) we hypothesized that ß2AdR dysfunction could be of critical importance in the pathophysiology of ME/CFS [43], see Textbox 1). We described a disturbed interaction between the cardiovascular system and the skeletal muscle, where dysfunction of vascular and cardiac ß2AdR together with endothelial dysfunction of other causes plays a key role.

Textbox 1 [43] **Pathophysiology of ß2-adrenergic receptor, vascular dysfunction and hypoxemia**

- ß2AdR dysfunction could be of critical importance in the pathophysiology of ME/CFS potentially caused by dysfunctional autoantibodies against ß2AdR, polymorphisms and desensitization of ß2AdR by (chronic) high sympathetic tone.
- Endothelial or vascular dysfunction, to which dysfunctional ß2AdR contributes, together with a high sympathetic tone may lead to an excessive vasoconstrictor stimulus in skeletal muscles.
- Muscular hypoperfusion stimulates generation of endogenous algesic vasodilators as a compensatory mechanism that is strongly favored by the poor background energetic situation in skeletal muscle which itself is due to a still enigmatic metabolic disturbance (probably a mitochondrial disturbance) [15].
- Spillover of such excessively generated algesic vasodilators into the circulation could explain the cardiovascular situation causing hypovolemia, mainly by the renal effects, the renin paradoxon (low renin) and preload failure further raising sympathetic (vasoconstrictor) tone.
- The appearance of algesic vasodilators in the systemic circulation may explain many of the enigmatic symptoms of ME/CFS including pain, hyperalgesia, intestinal complaints, flu-like symptoms, edema and sore lymph nodes.

In a second paper we tried to explain the potential causes of the energetic disturbances in skeletal muscle, where again, dysfunctional ß2AdR could play a key role via the need for ß2AdR-mediated stimulation of the Na^+^/K^+^-ATPase during exercise, which is a critical transport system for skeletal muscle metabolism [44].

Textbox 2 [44] **Pathophysiology of skeletal muscle disturbances**

- ß2AdR stimulates the Na^+^/K^+^-ATPase in skeletal muscles. Appropriate muscular perfusion as well as function of the Na^+^/K^+^-ATPase determine muscle function.
- Dysfunction of ß2AdR leads to an insufficient stimulation of the Na^+^/K^+^-ATPase causing sodium overload which reverses the transport direction of the sodium-calcium exchanger (NCX) importing calcium instead of exporting.
- The ensuing calcium overload affects the mitochondria, cytoplasmatic metabolism and the endothelium which further worsens the energetic situation.
- In poor energetic situations increased proton production raises intracellular sodium via the sodium-proton-exchanger subtype-1 (NHE1) in skeletal muscle.
- Calcium overload and impaired energy metabolism explain postexertional malaise, exercise intolerance and chronification.

The aim of the present hypothesis paper is to propose causes of the frequent neurological symptoms in ME/CFS, and how the previously identified pathomechanisms could also translate into the neurological symptoms, that we have not addressed so far. In our unifying hypothesis of the pathophysiology of ME/CFS and the pathophysiology of the skeletal muscle disturbances [43, 44] there is no need to assume a specific neurological pathology to explain the neurological symptoms. These are sufficiently explained by a stringent application of the ideas on the pathomechanisms already put forward in our previous two hypothesis papers. In the following we will discuss in detail how they could affect the brain to produce the various neurological symptoms.

## Decreased cerebral blood flow (CBF)

In the first two hypothesis papers we identified ß2AdR dysfunction, high sympathetic tone (stress), hypovolemia (preload failure), endothelial or vascular dysfunction, and an energetic disturbance in skeletal muscle as the main critical factors in the ME/CFS pathophysiology [43]. Meanwhile more evidence for endothelial dysfunction has accumulated. Altered endothelial dysfunction-related microRNAs were found in plasma from ME/CFS patients recently [5]. In Covid-19 infection the endothelium is severely affected and the disturbance seems to persist in post-Covid Syndrome (PCS) [12, 16, 24, 27, 38]. Particularly the finding of severe endothelial affection in Covid-19 and the persisting endothelial dysfunction corroborates our hypothesis of a role of endothelial and vascular dysfunction in the pathogenesis of PCS. Endothelial dysfunction may result in decreased CBF.

Patients with ME/CFS have a reduced absolute CBF [46]. Another very consistent finding is a decrease in CBF during orthostatic challenge (head-up tilt testing), i.e. after leaving the horizontal position. End-tilt CBF reduction was 7% in healthy controls versus 26% in ME/CFS, and a 24.5% decrease was decribed already after changing the position from supine to sitting in severe ME/CFS [7–10]. As a possible explanation for the orthostatic intolerance and the decrease in CBF we assume the presence of both a strong vasoconstrictor effect mediated by an elevated sympathetic tone and weakened vasodilator influences, that occurred in particular by dysfunction of ß2AdR and other causes of endothelial and vascular dysfunction. Covid-19 seriously affects the endothelium and there is evidence of chronic endothelial dysfunction in the post-Covid-syndrome similar to that in ME/CFS [12]. ß2AdRs have vasodilator effects in the brain, skeletal muscle and the heart, a mechanism to increase blood flow during muscular activity (a functional unit with the brain steering and coordinating muscle activity and the heart providing the blood flow for both organs). Since orthostatic stress is permanent during human activities (standing and even sitting) it is the basic stressor to which all other forms of stress are additive. It can desensitize ß2AdR and cause vasoconstrictor predominance by the α1-adrenergic effects that do not desensitize upon chronic stimulation in contrast to ß2AdR [43]. Chronic psychosocial stress itself can cause endothelial dysfunction and an increase in vasconstrictor mechanisms by functional and structural changes fixing the state of vasoconstriction (similar to the mechanisms that lead to fixed hypertension) [23, 47]. Endothelial dysfunction, which is clearly present in ME/CFS and PCS, has been found associated with cognitive impairment in different conditions, in the elderly as well as in children [17, 41]. Since the decrease in CBF already occurs after changing position from horizonal to sitting, orthostatic stress can be considered as the basic and permanent stressor during human activities in the awake state. We think that a decrease in CBF by 25% already in a sitting position, a concomitantly disturbed neurovascular coupling and endothelial dysfunction will not allow enduring cognitive efforts or mental work, with mental fatigue being the consequence.

Psychomotor slowing, ataxia and loss of coordination of movements in ME/CFS [11, 39] can also be explained by reduced perfusion, hypoperfusion of the motorcortex and other structures involved in motor function not being able to maintain coordinated neuronal activity in the motor cortex, a disturbance similar to impaired cognition. Since cognitive as well as motor function are disturbed by reduced (global) CBF there is good reason to believe that other brain functions can also be disturbed as will be explained in the following.

## Disturbed local blood flow regulation and neurovascular coupling

A reduction of global CBF is not the only cerebrovascular finding in ME/CFS. Consistent observation of sluggish fMRI signals suggests abnormal neurovascular coupling meaning that local blood flow regulation is also disturbed [32, 33, 37]. Endothelial dysfunction may play a role in the disturbance of neurovascular coupling. The question is whether the inflammatory mediators released from skeletal muscle can also affect neurovascular coupling, the local regulation of blood flow adjusting local perfusion to local neurophysiological activitiy. We think that influx of vasodilators, which otherwise also regulate local perfusion in other organs, into the cerebrovascular bed being under excessive vasoconstrictor influences via heightened sympathetic tone (stress), dysfunction of ß2AdR and endothelial dysfunction can only disturb a highly regulated fine tuning of vascular regulation and local blood distribution (neurovascular coupling).

## Increase in intracranial presssure

A more recent finding is an increase in intracranial presssure based on neuroradiological signs in an MRI study. Signs of intracranial hypertension (IH) can be shown in 83% of the patients with ME/CFS [6]. A further argument for a raised intracranial pressure is a higher prevalence of perineural or Tarlov cysts in the spinal cord in ME/CFS patients compared with healthy people [20]. These cysts are nerve root dilations resulting from pathologically increased cerebrospinal fluid pressure. They initially affect sensory neurons and axons in dorsal root ganglia and produce sensory symptoms consistent with paresthesias and radicular pain as does the increased cerebrospinal pressure itself. IH can also be a cause of headache [18]. A rise in intracranial pressure is at the expense of cerebral perfusion pressure which may have the strongest effect at the level of capillary blood flow. Possibly for this reason, IH is also associated with cognitive impairment [18].

Hypoperfusion of skeletal muscle together with mitochondrial dysfunction leads to the excessive production of various endogenous vasodilators in skeletal muscles and to their spillover into the systemic circulation, from where they can reach every organ including the brain [43]. One of these mediators, bradykinin, is the most potent opener of the blood brain barrier (BBB) [1, 31] which may be of relevance for the neurological findings and symptoms. Opening of the BBB may explain moderate IH for which we have provided the evidence above. We could not find in the literature what the symptoms of isolated opening of the BBB would be but we assume it to be rather pathological. The algesic and hyperalgesic properties of the tissue mediators released from skeletal muscles may cause headache by directly acting on cerebrovascular nociceptors, by the release of CGRP and substance P and by edematous distension. Headache could also originate from myalgia of head and neck muscles and from IH [18].

## Disturbances of reflexes and autonomic function, hypervigilance and hypersensitivity to sensory stimuli such as light, noises and smells and brain fog

Disturbances of the pupillary reflex [4] upon prolonged illumination of the pupils were also reported in patients with ME/CFS: “Two unusual responses were observed that are evident on prolonged illumination of the pupils. The more frequent finding seen in three quarters of patients is a rhythmic contraction and dilatation of the pupils. The second pattern is a paradoxical dilation of the pupils after an initial contraction.“ Thus, one should consider the possibility that also other regulatory mechanisms of body and autonomic functions and reflexes, like orthostatic regulation, vascular regulation, thermoregulation and sleep are also impaired as a consequence of a reduced CBF. This could result in either sluggish responses to sudden changes or the opposite, namely large overshooting swings unable to find a new stable level of regulation after a disturbance, similar as observed with the pupillary reflex. In such a way impaired regulation of orthostatic function may contribute to orthostatic dysregulation and intolerance without necessarily being the main cause of it. Hence, dysautonomia may be enhanced by a disturbed CBF. Primary disturbances of the autonomic nervous system and dysautonomia or autonomic dysfunction arising from the brain stem may play a role in triggering ME/CFS for instance by orthostatic dysfunction causing orthostatic stress that would desensitize ß2AdR and raise α-adrenergic mediated vasoconstriction. Based on these considerations, it should be considered that ME/CFS itself could cause or at least worsen autonomic dysfunction by cerebral hypoperfusion from which another vicious circle would arise. By these mechanisms autonomic dysfunction could also be expanded to other primarily undisturbed autonomic functions.

Neuroinflammation of the brain stem has been discussed as a possible cause of dysautonomia but no clear evidence has been provided so far [40]. If neuroinflammation of the brain stem was the primary cause of dysautonomia the question would still have to be answered of how such a disturbance could cause the myriad of symptoms and objective findings like muscle weakness, pH changes in skeletal muscles, PEM and cardiovascular findings like hypovolemia with low RAAS activity. Our “unifying” hypothesis allows to explain how dysautonomia independent of its primary cause can cause the myriad of symptoms and enigmatic findings of ME/CFS.

Hypervigilance and sympathetic hyperactivity are present in ME/CFS [14]. Stress may cause hypervigilance by centrally stimulatory effects of catecholamines and PGE_2_ (one of the mediators released into the circulation by the skeletal muscles). The latter has awakening effects [19]. The α2-adrenergic autoreceptor, a presynaptic receptor on noradrenergic neurons modulating and inhibiting norepinephrine release [30] shows desensitization [3] similar to the ß2AdR, but unlike α1-adrenergic receptors. Thus, chronic stress could also desensitize this inhibiting autoreceptor to enhance catecholamine release in activating noradrenergic nuclei of the brain stem like the locus coeruleus thereby causing arousal and increased vigilance, and in the sympathetic nervous system leading to enhanced vasoconstrictor output while the vasodilator ß2AdR is dysfunctional and endothelial dysfunction of other causes may be present. It would finally mean that chronic stress enhances stress responses (adrenergic hyperactivity). Orthostatic stress may play a particular role since it is already present in the sitting position in ME/CFS to decrease CBF and therefore almost unavoidable. Since orthostatic stress is operative over a considerable part of the day there is not sufficient time for recovery of desensitized adrenergic receptors. Episodes of stress in human life are usually followed by episodes of rest and recovery in which re-sensitization of adrenergic receptors can take place and therefore such enhancement of stress responses can fade if sufficient time of recovery is allowed (presumeably only few days of rest needed for receptor re-sensitization). In line with these considerations, long and intense periods of psychosocial stress, in which such desensitization could take place, precede the development of ME/CFS in a subset of patients which is a potential explanation for the initiation of the disease in this subset. By the mechanisms outlined in our previous paper, ME/CFS, once fully established, is a state in which a high level of stress is maintained and fixed by a number of dysregulations and vicious circles, which the patient can hardly escape [44]. Since there is no recovery from stress in ME/CFS, α2-adrenergic autoreceptors and ß2AdR remain desensitized.

Possible desensitization of the α2-adrenergic receptor (α2AdR) located in the noradrenergic nuclei and in cardiovascular centers of the medulla oblongata should be added to the possible brainstem pathomechanisms involved in dysautonomia. Dysfunction of the α2AdR autoreceptor could cause overshooting sympathetic responses by enhanced catecholamine release that may contribute to orthostatic dysregulation, cause disturbances of respiratory control (hyperventilation) and affect other autonomic functions.

Hypervigilance caused in the way just described may lead to increased stimulus uptake in all sensory organs while stimulus processing in the corresponding brain regions may be impaired by the reduced CBF as outlined above. Increased stimulus uptake and concomitantly reduced stimulus processing may lead to stimulus overload. Stimulus overload could be the cause of hypersensitivities against sensory stimuli such as light, noises and smells.

These considerations also help to understand the simultaneous presence of signs of hypervigilance and mental fatigue in ME/CFS patients. This paradox can be explained by the discrepancy between the higher energy demand caused by neuronal overstimulation and the reduced energy supply by cerebral hypoperfusion resulting in an early energetic deficit to explain the high level of mental fatigability. The ability to perform mental work may be additionally diminished by hypervigilance (nonspecific overstimulation) reducing the ability to concentrate on a single mental task which may be part of the mechanisms causing the feeling of brain fog. Finally, brain fog may be the result of central overstimulation, cerebral hypoperfusion, IH and opening of the BBB allowing the endogenous mediators released from skeletal muscle like bradykinin and PGE_2_ to exert effects in the brain.

## Hypocapnia, hyperventilation, respiratory alkalosis and possible consequences for skeletal muscle metabolism

A frequent finding in ME/CFS and postural tachycardia syndrome (POTS), which is frequently associated with ME/CFS, is hypocapnia and hyperventilation [14, 39]. Hypocapnia causes cerebral vasoconstriction enhancing the effect of cerebral vasoconstriction induced by the sympathetic nervous system. In POTS it was shown that there is no fall in blood pressure,thus brain perfusion pressure is maintained [35]. Cerebral blood flow velocity of the middle cerebral artery was reduced during head‐up tilt associated with hyperventilation. Thus the decrease in CBF can be explained only by a rise in cerebrovascular resistance by vasoconstriction.

We consider hyperventilation, an overshooting response to a respiratory stimulus, resulting from autonomic dysfunction with sympathetic overactivity. In PCS hyperventilation has recently been identified as cause of dyspnea. Hyperventilation occurred early during exercise resulting in an impaired ventilatory efficiency [2, 26]. The cause may be excessive stimulation of the respiratory center in the brain stem causing the feeling of air hunger (dyspnea). A physiological early mechanism of respiratory stimulation is via skeletal muscle afferents involving movement sensors and metabolic afferents [34]. It is obvious to incriminate a disturbed metabolic situation in skeletal muscle in this early, excessive stimulation of respiration during exercise. The latter could then be further enhanced by dysautonomia (as an overshooting response). The resulting dyspnea is not only limiting the ability to exercise but aggravates the key pathophysiological mechanisms in skeletal muscle in ME/CFS. Hyperventilation induced respiratory alkalosis is buffered by a shift of protons from the intracellular to the extracellular space mainly via the NHE1 thereby loading the cells with sodium. Sodium overload in ME/CFS reversing the transport mode of the NCX to cause calcium overload is therefore not only the consequence of an insufficient Na^+^/K^+^-ATPase-activity and a glycolytic metabolism but hyperventilation may contribute and worsen it. Altogether, dysautonomia together with the disturbed skeletal muscle metabolism may cause hyperventilation and respiratory alkalosis—at least during exercise—and thereby further disturb the skeletal muscle metabolic situation by sodium- and calcium- overload (vicious circle).

## Sleep disturbances and non-restorative sleep

Difficulties to fall asleep and altered day-night rhythm may result directly from enhanced catecholamine release in activating noradrenergic nuclei of the brain stem like the locus coeruleus as a consequence of desensitized α2-autoreceptors and stress causing arousal and increased vigilance. Stimulus overload just explained above could be an indirect cause of sleep disturbances. In sleep medicine it is considered an important cause of insomnia.

Sleep quality may also be affected by disturbed nocturnal breathing and respiratory disturbances. Hypocapnia and hyperventilation are frequently found in ME/CFS during wakefulness [14, 39]. Patients with whiplash injury, who frequently suffer from ME/CFS, also present with hyperventilation [43]. Hypocapnia as a consequence of hyperventilation has destabilizing effects on breathing during sleep to mainly cause central apneas (due to the existence of the CO_2_-apneic threshold) [42]. The key question is whether hyperventilation and hypocapnia also occur during sleep in ME/CFS which has not yet been investigated. It is rather unlikely that a respiratory disturbance present in the awake state at rest will disappear with sleep onset. This is because the so-called wakefulness drive to breathe has a stabilizing effect so that breathing usuallys gets even more unstable during sleep [13]. Even mild sleep-related breathing disorders in ME/CFS would have the potential to make sleep another big stressor and to severely affect the general health condition of ME/CFS patients. Since the destabilizing effect of hyperventilation and hypocapnia on nocturnal breathing is strong and well established [13] and since the consequences of intermittent hypoxia with the ensuing sympathetic surges could be much more severe in ME/CFS compared with sleep apnea patients, the negative consequences could even appear at lower levels of hypoxia and oxygen desaturation. Due to the predominance of vasoconstrictor over vasodilator influences sympathetic surges arising from intermittent hypoxia in the post-apneic phase would probably much more severely affect perfusion of the brain and skeletal muscles in patients with ME/CFS than in patients with sleep apnea only. During sleep this could lead to the compensatory release of endogenous algesic vasodilators from skeletal muscles and spillover into the systemic circulation as already postulated for exercise and stress situations in the awake state. This could further disturb sleep since PGE_2_ has awakening effects [19]. It could also further disturb nocturnal breathing as upper and lower airway (bronchial) resistance may be raised due to edema (microvascular leakage) and the spasmogenic effect of mediators like PGE_2_ and bradykinin in the bronchi.

Poor sleep can certainly worsen fatigue and symptoms in ME/CFS, but even a perfect sleep could not abolish mental and skeletal muscle fatigue because the main causes of fatigue are unrelated to sleep. Mental and muscular fatigue are related to insufficient blood flow and, concerning skeletal muscle fatigue, there is an additional energetic disturbance, namely mitochondrial dysfunction. At least the term “non-restorative sleep “ should therefore be used with caution and in the knowledge that disturbed sleep may worsen fatigue but it is not the main cause of it.

## Conclusion

**Fig. 1:**
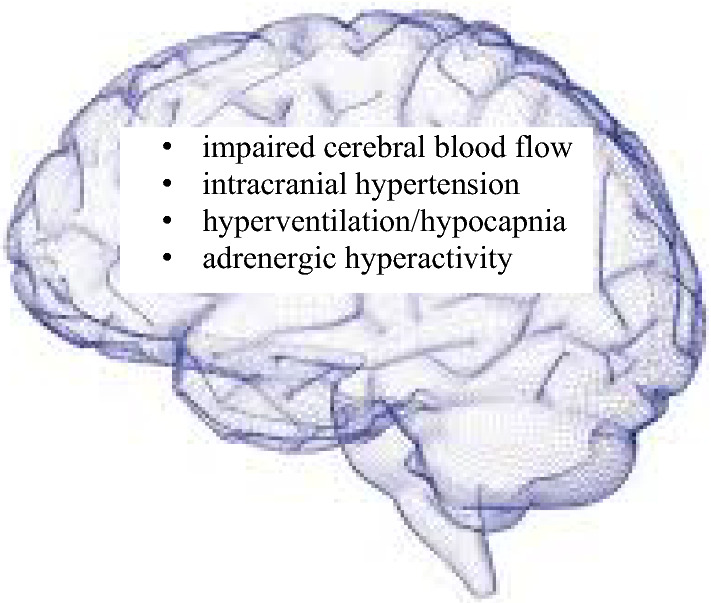
Key neurological pathomechanisms in ME/CFS

Neurological symptoms in ME/CFS can be severe and debilitiating, but no clear specific brain pathology or lesions have been detected so far. Whether neuroinflammation or a brain stem pathology exists—where dysautonomia may have its origin as the primary disturbance eliciting ME/CFS—remains to be shown [40]. Decreased CBF, disturbed local blood flow regulation and neurovascular coupling, central adrenergic hyperactivity, hypocapnia and increase in intracranial presssure seem to play a strong role in the pathophysiology of the neurological symptoms in ME/CFS (Fig. 1). They can well explain cognitive impairment, brain fog, headache, psychomotor slowing, ataxia and loss of coordination of movements, hypersensitivity, sleep disturbances and dysautonomia.

## Data Availability

Not applicable.

## Abbreviations

α_2_AdR: α_2_-Adrenergic receptors
ß_2_AdR: ß_2_-adrenergic receptors
BBB: Blood brain barrier
CBF: Cerebral blood flow
IH: Intracranial hypertension
ME/CFS: Myalgic Encephalomyelitis/Chronic Fatigue Syndrome
PEM: Post-exertional malaise
POTS: Postural tachycardia syndrome
PCS: Post-Covid-19 syndrome
RAAS: Renin–angiotensin–aldosterone system
NCX: Sodium-calcium exchanger
NHE1: Sodium-proton-exchanger subtype-1
