# Correction to: An attempt to explain the neurological symptoms of Myalgic Encephalomyelitis/Chronic Fatigue Syndrome

**DOI:** 10.1186/s12967-021-03216-3

**Published:** 2022-01-11

**Authors:** Klaus J. Wirth, Carmen Scheibenbogen, Friedemann Paul

**Affiliations:** 1 KOSA Pharma GmbH, Frankfurt am Main, Germany; 2 Institute of Medical Immunology, Charité - Universitätsmedizin Berlin, Corporate Member of Freie Universität Berlin, Humboldt-Universität Zu Berlin, and Berlin Institute of Health, Berlin, Germany; 3 Experimental and Clinical Research Center, Max Delbrück Center for Molecular Medicine and Charité Universitätsmedizin Berlin, Berlin, Germany

## Correction to: Journal of Translational Medicine (2021) 19:471 https://doi.org/10.1186/s12967-021-03143-3

Two errors were noticed after publication of the original article [1]:

1. Carmen Scheibenbogen should also be a corresponding author
2. 4 references and their citations were missing [2–5] in the Decreased cerebral blood flow (CBF) section.

The original article has been updated to correct these errors.
